# P-391. Infections in Gender-Affirming Surgery: A Single-center Experience with Gender-affirming Vaginoplasty

**DOI:** 10.1093/ofid/ofae631.592

**Published:** 2025-01-29

**Authors:** Radhika Sheth, Apoorva Bhaskara, Haley M Brown, Cara D Varley, Amber C Streifel, Marissa Maier, Monica K Sikka, Christopher Evans

**Affiliations:** Henry Ford Health, Ann Arbor, Michigan; Oregon Health and Science University, Portland, OR; Oregon Health & Science University, Portland, Oregon; Oregon Health & Science University, Portland, Oregon; Oregon Health and Science University, Portland, OR; VA Portland Health Care System/Oregon Health and Sciences University, Portland, Oregon; Oregon Health and Science University, Portland, OR; Oregon Health and Science University, Portland, OR

## Abstract

**Background:**

There are an estimated 1.3 million transgender adults in the US and about 25% undergo some form of gender-affirming surgery (GAS). Data on infectious complications following GAS is limited. We describe the epidemiology and incidence of infections following gender-affirming vaginoplasty (GAV). We also describe HIV screening and pre-exposure prophylaxis (PrEP) use in this population as transwomen are disproportionately affected by HIV and have the highest prevalence compared to any other group of US adults.

**Figure:**
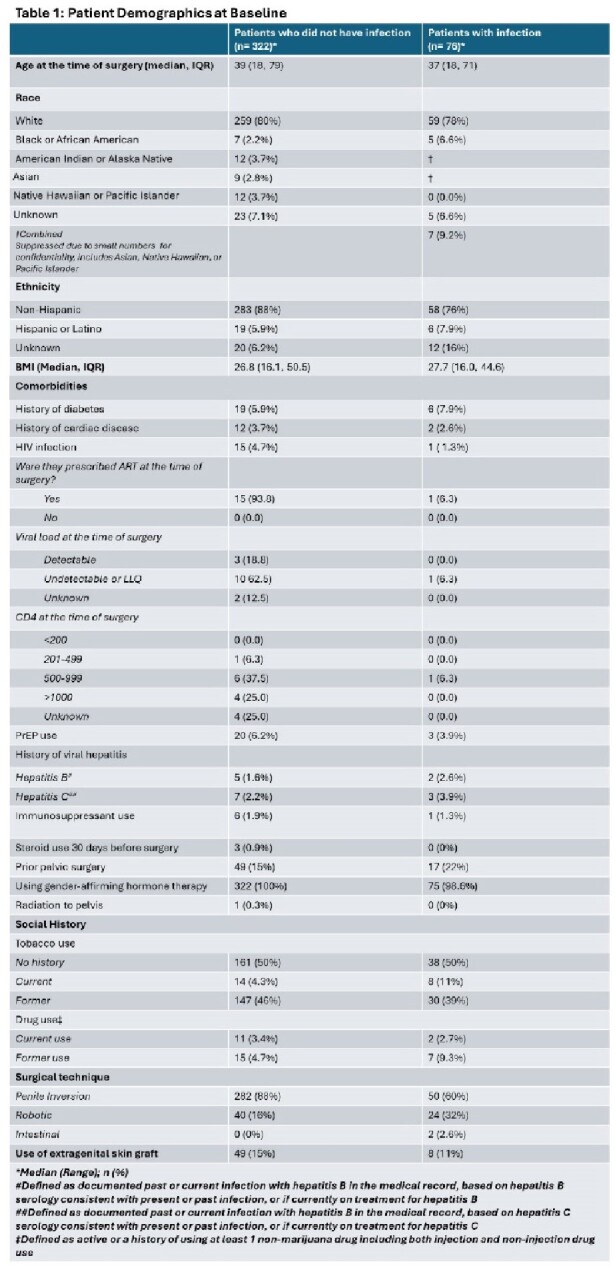

**Methods:**

In this single-center retrospective cohort study, we identified 398 patients aged ≥18 years who underwent GAV at Oregon Health & Science University from 2016 to 2023. We reviewed medical records up to 6 months from the initial surgery and used standardized criteria to diagnose surgical site infections (SSI), urinary tract infections (UTI), and sexually transmitted infections (STI). Data around HIV screening and PrEP use was also obtained.

**Figure:**
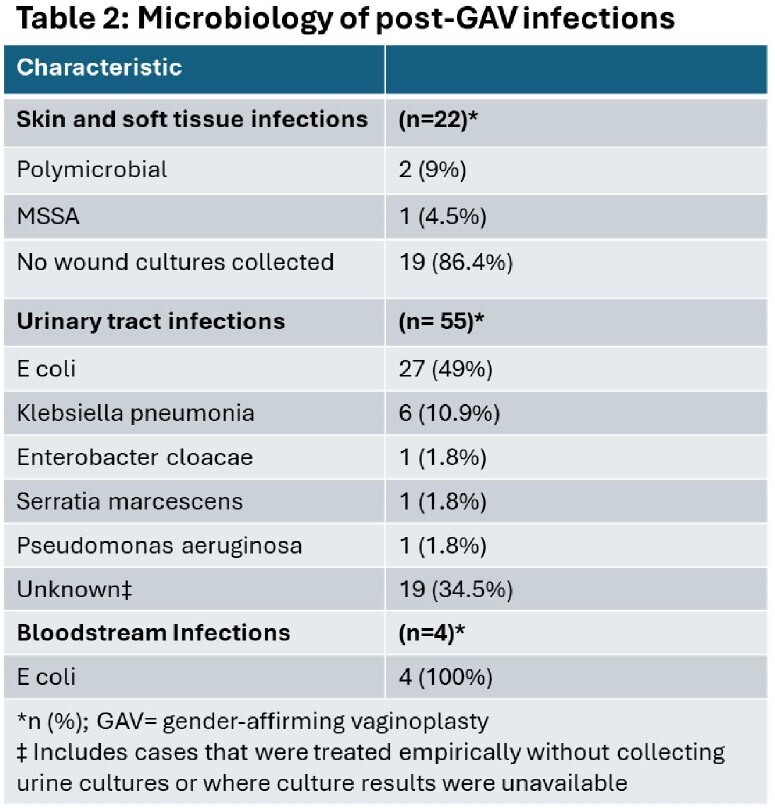

**Results:**

381 patients underwent primary vaginoplasty and 17 underwent revision of the primary surgery. 332 had standard penile inversion, 64 had robotic vaginoplasty, and 2 patients had intestinal vaginoplasty. UTI was the most common postoperative complication (incidence 13.8 per 100 individuals; n=55), followed by SSI (incidence 5.5 per 100 individuals; n=22). We identified 4 episodes of bacteremia related to surgery- all due to *E coli*- and one case of pelvic abscess. Only 2 STIs were identified and neither involved the neogenitalia- 1 was primary syphilis and the other was gonococcal urethritis.

16 patients had positive HIV serostatus. All 16 were prescribed ART and 11/16 had an undetectable viral load at the time of surgery. Only 22.5% of HIV seronegative patients had screening within a year before surgery and only 6% were on PrEP at the time of surgery.

**Figure:**
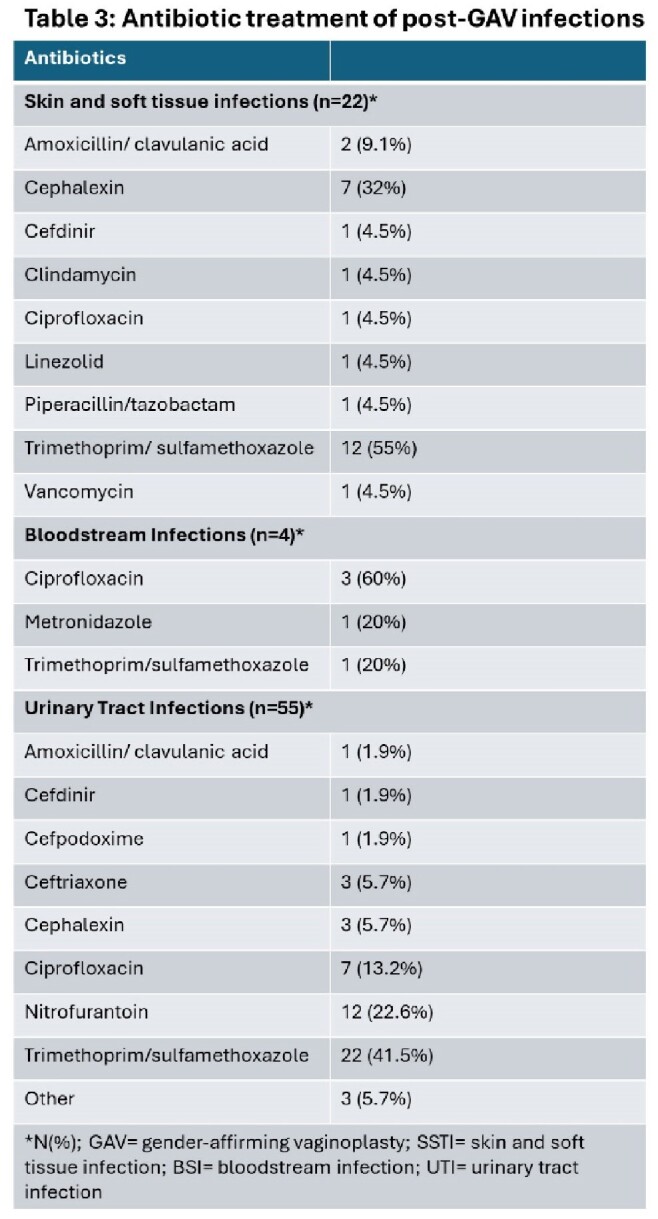

**Conclusion:**

In our study, UTIs and SSIs were the most common infections post-GAV. Serious infections were rare.

We also identified a significant gap in HIV screening and PrEP uptake in this especially vulnerable group. Transgender surgery programs should incorporate HIV screening and counseling around PrEP.

**Disclosures:**

**Monica K. Sikka, MD**, F2G: Grant/Research Support **Christopher Evans, MD**, ViiV: Grant/Research Support

